# Beyond natural: synthetic expansions of botanical form and function

**DOI:** 10.1111/nph.16562

**Published:** 2020-04-23

**Authors:** Nicola J. Patron

**Affiliations:** ^1^ Engineering Biology Earlham Institute Norwich Research Park, Norwich Norfolk NR4 7UZ UK

**Keywords:** bioengineering, metabolic engineering, plant biotechnology, plant synthetic biology, protein engineering

## Abstract

Powered by developments that enabled genome‐scale investigations, systems biology emerged as a field aiming to understand how phenotypes emerge from network functions. These advances fuelled a new engineering discipline focussed on synthetic reconstructions of complex biological systems with the goal of predictable rational design and control. Initially, progress in the nascent field of synthetic biology was slow due to the *ad hoc* nature of molecular biology methods such as cloning. The application of engineering principles such as standardisation, together with several key technical advances, enabled a revolution in the speed and accuracy of genetic manipulation. Combined with mathematical and statistical modelling, this has improved the predictability of engineering biological systems of which nonlinearity and stochasticity are intrinsic features leading to remarkable achievements in biotechnology as well as novel insights into biological function. In the past decade, there has been slow but steady progress in establishing foundations for synthetic biology in plant systems. Recently, this has enabled model‐informed rational design to be successfully applied to the engineering of plant gene regulation and metabolism. Synthetic biology is now poised to transform the potential of plant biotechnology. However, reaching full potential will require conscious adjustments to the skillsets and mind sets of plant scientists.

1

**Table:** 

	Contents	
	Summary	295
I.	Introduction	295
II.	Applying the principles of engineering to biology	296
III.	Engineering gene regulation	301
IV.	Engineering proteins	303
V.	Engineering metabolism	304
VI.	Emerging research areas	305
VII.	Applications and prospects	306
	Acknowledgements	307
	References	307

## I. Introduction

Similar to other emerging fields, synthetic biology has struggled to define itself to outsiders. Perhaps the most visible aspects have been impactful tools and techniques such as large‐scale DNA assembly, genome engineering and cell‐free protein expression. Thus, there has been a tendency for biologists to perceive synthetic biology as a field focussed on the development and application of novel technologies, while the media typically report it as ‘advanced biotechnology’. However, synthetic biology self‐defines as an emerging discipline of engineering. At its core is the central theory that applying engineering principles and approaches improves the predictability and increases the ease and efficiency with which biological systems can be designed, constructed, and characterised (Chen *et al.*, 2012). It has long been noted that engineers tackle problems by systematically applying formal approaches and employing computational power to make complex problems tractable. It is therefore argued that if biologists were to adopt formal descriptions of biological processes, this would render cells less complex and more accessible. A particularly convincing argument was made by comparing the random approaches used by biologists to the systematic approaches used by engineers to identify a faulty component in a malfunctioning system (Lazebnik, 2002).

**Fig. 1:**
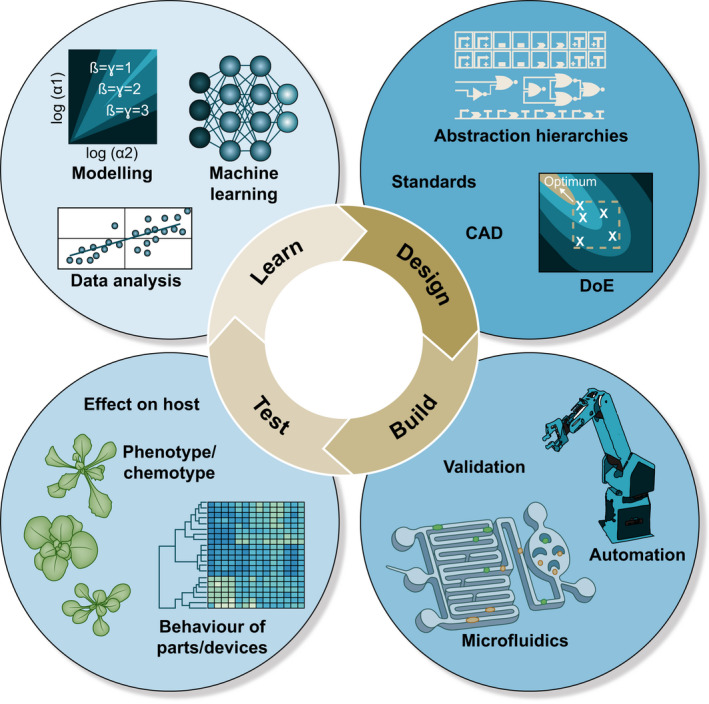
Synthetic biology employs iterative cycles of design, build, test and learn. The data generated in each turn of the cycle are used to improve models of the system and inform the next cycle. Design is facilitated by the use of standards and abstraction hierarchies, Computer Aided Design (CAD) and the systematic application of statistics design of experiments (DoE), enabling large‐scale experiments. Such experiments employ laboratory automation and microfluidics to increase reproducibility, minimise reaction volumes and reduce errors. Statistical and computational techniques are used to analyse data and refine predictive models.

The goal of synthetic biology is to advance the ability to dependably and consistently design or reprogramme living organisms and to fabricate products from biologically derived materials. The route to achieving this is predicted to lie in the application of mathematical methods, the adoption of standardisation and modularity, and the use of abstraction hierarchies to manage biological complexity during the design process (analogous to the use of higher‐level programming languages in computing). These approaches are applied in iterative cycles of design–build–test–learn, where quantitative data on performance are fed into predictive models to improve later design cycles (Fig. 1). The application of engineering approaches is considered to be essential if biotechnology is to realise its ambition of enabling a new industrial revolution able to rival the flexibility and scales of manufacturing currently achieved through chemical (particularly petrochemical) and electronic engineering (Endy, 2005). Adopting these foundational principles has provided the synthetic biology research community with both self‐identity and a set of common goals on which early efforts were heavily focussed (Arkin, 2008; Canton *et al.*, 2008).

It should be noted, however, that there is not total acceptance that synthetic biology is able to operate as a field of engineering (Kwok, 2010; Davies, 2019). Engineering typically begins with a clearly defined goal, followed by a careful design phase that uses well defined models together with measurable and described methods to predict the behaviour of components in the combinations in which they will be used. By contrast, our incomplete understanding of biology hinders our ability to fully adopt the outlooks and working practices of engineering. While there has undoubtedly been significant progress, particularly in the application of metabolic modelling to improve yields in biomanufacturing (King *et al.*, 2015), synthetic biology projects must, necessarily, combine experimental investigations of biological function with model‐informed engineering.

**Fig. 2:**
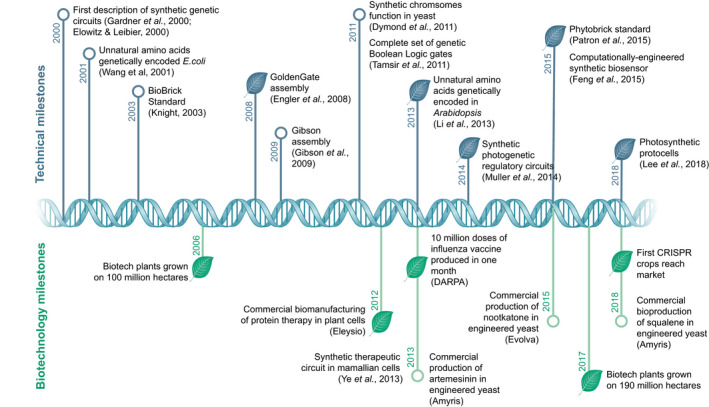
Key milestones in synthetic biology and biotechnology. Leaf symbols denote milestones in plant and plant‐related systems, open circles denote milestones in nonplant systems.

Progress in plant synthetic biology has lagged behind the microbial field partly due to practical problems posed by lengthier life cycles and the additional complexities of working with larger genomes and multicellularity. Arguably, the wider plant science community has also been relatively slow to adopt synthetic biology approaches into wider research practice. Nevertheless, plant synthetic biology is following a similar trajectory to that seen in the microbial field in the first decade of the 21^st^ century (Fig. 2). Foundational engineering principles such as community standards have been developed (Patron *et al.*, 2015; Zhao & Medema, 2016) and, simultaneously, there have been impressive advances in the application of computational modelling to plant growth and metabolism (for recent reviews see Gomes de Oliveira Dal'Molin & Nielsen, 2018; Morris, 2018). In recent years, engineering strategies informed by mathematical models have led to remarkable successes in increasing plant biomass and modulating plant responses to environment (Park *et al.*, 2015a; South *et al.*, 2019; Vaidya *et al.*, 2019). Similarly, the development of tools such as biosensors to aid functional quantification of metabolic signals has enabled rational manipulations of plant growth and development (Drapek *et al.*, 2018; Khakhar *et al.*, 2018).

**Fig. 3:**
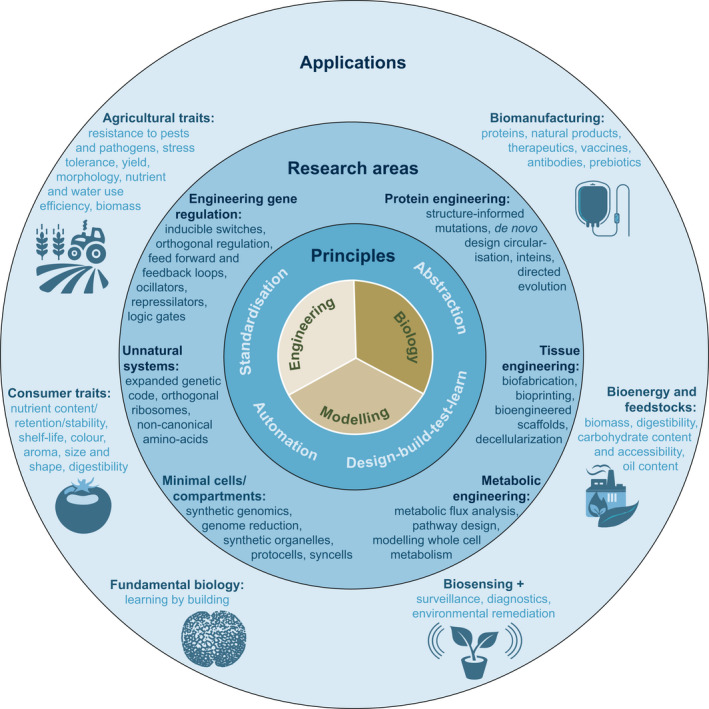
Focus areas in plant synthetic biology. Synthetic biology lies at the intersection of engineering, biology and mathematical modelling (centre). The central theory is that the application of engineering principles (second ring) will facilitate the engineering of biological systems and materials. These are applied in diverse research areas (third ring) with the aim of improving the predictability with which they can be engineered. Multiple strategies are combined to develop new knowledge and products for the bioeconomy (outer ring).

Plant synthetic biology encompasses diverse research areas, each focussed on improving the predictability of a particular approach to reprogramming biological systems through the application of engineering principles (Fig. 3). These approaches are applied, often in combinations, to advance biotechnological aims, but also to investigate fundamental biological questions (Fig. 3). As a result, the plant synthetic biology community is populated by researchers taking conceptually similar experimental approaches to problems as diverse as the rational design of proteins with novel functions; *in vitro* construction of tissues from living cells; assembly of robust regulatory networks; and the creation and testing of protocells to investigate biophysical processes and the origins of life (Fig. 3). These researchers tend to utilise different meanings of the word ‘synthetic’: Those working with nonevolved features such as the expansion of the genetic code with noncanonical amino acids tend to use synthetic as a synonym for ‘artificial’ or ‘unnatural’ while those aiming at the production of proteins and metabolites tend to use the more ancient meaning of synthetic as a product of synthesis (synthesis being derived from the Greek ‘*suntithenai*’, meaning ‘to put together’).

Recent reviews have examined how crop‐improvement or the production of biofuels and plant natural products might be advanced by applying synthetic biology approaches (Fesenko & Edwards, 2014; Shih *et al.*, 2016; Wurtzel *et al.*, 2019). By contrast, this text will consider progress in the development of engineering principles and foundations for biological design and will then review their application to the various approaches and technologies used to engineer plant systems. While progress in biotechnology will be discussed, the main aim of this manuscript is to highlight how synthetic biology approaches are impacting plant science. Although plants are the primary focus, the state‐of‐the‐art in other systems will also be outlined with the aim of inspiring plant scientists and predicting future directions of travel.

## II. Applying the principles of engineering to biology

At the core of engineering approaches are iterative cycles of design, build, test and learn in which the data generated are used to improve the next cycle of design (Fig. 1). This section will consider the development and application of engineering foundations, particularly those used in the design segment of the cycle. In molecular biology, which underpins much of biotechnology, design and build often comprise the selection and assembly of multiple fragments of DNA. Therefore, it was to this process that standardisation was first applied. For several decades, the assembly of DNA was largely limited to inserting single fragments of DNA into polylinkers (multiple cloning sites) of plasmids comprising recognition sites for various type II restriction endonucleases. For plants, the fragments were most often coding sequences inserted into the cloning sites of binary vectors flanked by regulatory sequences derived from Cauliflower Mosaic Virus or the opine biosynthesis genes from *Agrobacterium tumefaciens* (Komori *et al.*, 2007). The inclusion of additional features or the replacement of existing sequences was complicated and laborious. Moreover, the presence of different restriction endonucleases recognition sites within specific sequences meant that different sequence fragments might be most‐easily cloned into any site within the polylinker. Thus, the final sequence of junctions often differed between constructs. As these cloning junctions were usually adjacent to the start of transcription or translation, assemblies were often not functionally comparable. While this was of little importance when the experimental goal was simply to overexpress a given protein, it limited the quantitative comparison of, for example, regulatory elements. Products such as the Gateway^®^ cloning system (Thermo Fisher, Waltham, MA, USA) improved efficiency and flexibility. However, the recombinase‐enabled reaction did not allow for complex or precision design. Further, the proprietary nature was undesirable in the emerging synthetic biology community, which, inspired by progress in electronic and software engineering, prescribed that standards, infrastructure and enabling technologies should be ‘open’ in order to accelerate progress and provide an ecosystem encouraging of innovation and entrepreneurship (Calvert, 2012).

Engineers reason that, regardless of whether a system is built from parts machined from metal alloys or from fragments of engineered DNA, iterative improvements are vastly facilitated if construction is modular and components are standardised rather than bespoke. A commonly used metaphor is that of the screw: before the standardisation of screw heads and threads in the 1840s, a malfunctioning machine could be fixed only with considerable effort and only by the original manufacturer. Standardisation allowed repairs and improvements to be performed by third parties and enabled mechanics to collaborate on the delivery of large projects such railroads and fleets of ships. In the same spirit, the emerging synthetic biology community reasoned that the standardisation and modularisation of DNA parts would allow different sequences with the same basic utility to be exactly exchanged within larger designs enabling any differences in function conferred by sequence variations between parts to be functionally quantified (Arkin, 2008).

**Fig. 4:**
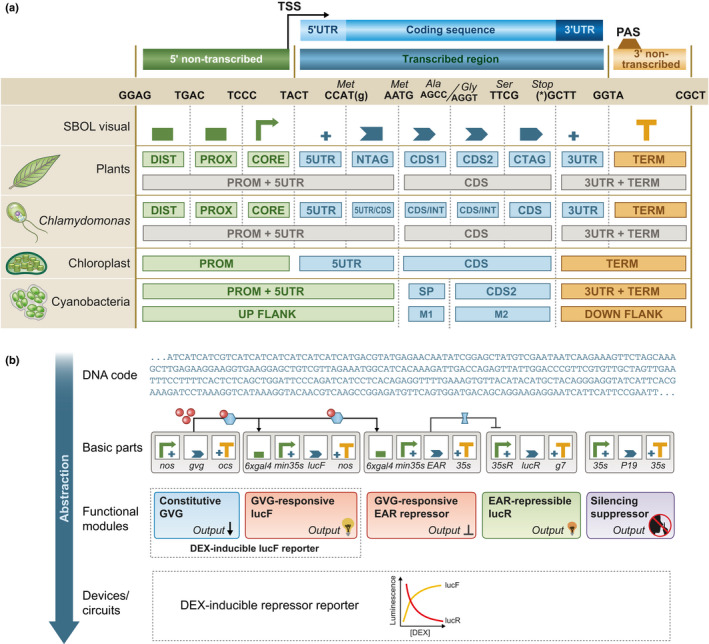
Summary of the Phytobrick standard and its application in abstraction hierarchies. (a) The Phytobrick syntax divides eukaryotic genes into 10 basic functional units: DIST (distal promoter region, *cis* regulator, or transcriptional enhancer); PROX (proximal promoter region or transcriptional enhancer); CORE (minimal promoter region, including transcription start site); 5UTR (5′ untranslated region); NTAG (amino‐terminal coding region); CDS1 and 2 (coding regions); CTAG (carboxy‐terminal coding region); 3UTR (3′ untranslated region); TERM (transcription terminator, including polyadenylation signal). Each part is represented by a Synthetic Biology Open Language visual (SBOL_v_) glyph. Phytobrick parts can comprise the region between an adjacent pair of fusion sites or span many sites. Each Phytobrick consists of portion(s) of a gene cloned into a plasmid flanked by a convergent pair of *Bsa*I Type IIS restriction endonuclease recognition sequences. Adaptations for other systems include the assignment of specific part types for use as flanking regions (up‐flank and down‐flank) to enable homologous recombination into the host genome, SP (signal peptide), and as selectable markers (M1 and M2). (b) A synthetic genetic circuit to measure simultaneous repression and activation in response to an external signal (dexamethasone; dex) can be described at four levels of abstraction: DNA level; graphical description of 17 Phytobricks; functional modules of five transcriptional units; a complete circuit for which activity could be quantitatively described.

The first biological standards were BioBricks (Knight, 2003), communicated through the newly established BioBricks Foundation Request for Comments process, an organisational framework that helps to define, evaluate and propose new standards in Synthetic Biology. BioBricks simplified the assembly of DNA using iterative, pairwise assembly of standardised parts. However, although BioBrick‐compatible binary plasmids were eventually constructed for plants (Boyle *et al.*, 2012), they were never widely adopted because new methods for simultaneous assembly of multiple DNA fragments were already available. These included methods such as Gibson Assembly, which assembles multiple linear, double‐stranded, overlapping fragments of DNA (Gibson, 2009), as well as those that use Type IIS enzymes, commonly known as GoldenGate assembly (Engler *et al.*, 2008). The development of Type IIS plasmid toolkits, for example ‘MoClo’ (Engler *et al.*, 2014), facilitated the assembly of multigene binary constructs for plants. With the aim of facilitating the exchange of interoperable, standardised DNA parts across the plant community, a so‐called ‘common genetic syntax’ (now known as the ‘Phytobrick’ standard) was established to define DNA parts for plants (Patron *et al.*, 2015; Fig. 4a). Subsequently, toolkits for engineering plastids (Occhialini *et al.*, 2019), algae (Crozet *et al.*, 2018), and cyanobacteria (Vasudevan *et al.*, 2019) have been based on this standard (Fig. 4a). Standardisation has since been cited as a facilitating factor in the progress of several plant biotechnology projects (South *et al.*, 2019; Ermakova *et al.*, 2020).

There have also been efforts to standardise experimental procedures for the characterisation of standard parts. It is argued that this is necessary to aid the rational selection of parts in new designs. In the microbial field, experimental standardisation has gained slightly more traction as it is relatively easy to define and reproduce growth conditions in which standardised measurements can be taken (Sainz De Murieta *et al.*, 2016). While similar benefits have been proposed for plant synthetic biology (Vazquez‐Vilar *et al.*, 2017), the requirement to characterise performance across cell types and over developmental time scales, coupled with the comparative complexity of standardising growth conditions has limited progress. Nonetheless, the argument that scientific data and materials should be shared in reusable formats in accessible repositories extends far beyond synthetic biology. To improve information on the design of synthetic constructs, the synthetic biology open language (SBOL), was established (Bartley *et al.*, 2015). SBOL also provides standards for the visual representation of genetic elements (Quinn *et al.*, 2015; Fig. 4a).

The use of standardised DNA parts directly enables abstraction hierarchies to be established. Once parts have been standardised, there is little need to observe the underlying DNA sequence: the sequence of the final assembly is determined by the identity of the selected parts and the assembly standard (Fig. 4b). This allows complex assemblies to be rapidly designed from a suite of known basic parts using sequence manipulation software including biology‐focussed Computer Aided Design (CAD) packages (described below). The process of abstraction enables engineers to focus on experiment design and the testing of unknowns such as functional quantification of novel parts, or how combinations of parts affect each other's function. For biologists, such advances may be of minor importance. However, for synthetic biologists aiming to improve the speed and process of design, the development of abstraction hierarchies is a route to enabling routine tasks like DNA assembly to be performed by nonspecialists or automated platforms. This provides for cost‐effective scaling, equivalent to the automated assembly of electronic components into complex circuitry according to designs provided by electronic engineers, or the assembly of steel and concrete by construction workers according to blueprints provided by civil engineers.

It follows, therefore, that there have been equivalent efforts in automating experimental workflows. The use of automated platforms in research laboratories had previously been relatively sparse, prohibited by high purchase and running costs and the requirement for technicians skilled in programming scripts. The use of automation to increase scale and reproducibility is considered to be essential to synthetic biology (Jessop‐Fabre & Sonnenschein, 2019). This is exemplified both by the emergence of synthetic biology‐sector companies focussed on software and hardware solutions for laboratory automation and by the emergence of biofoundries, facilities that specialise in automating the design–build–test–learn cycle (Chao *et al.*, 2017; Hillson *et al.*, 2019). Biofoundries enable experiments not possible using manual approaches, increasing the scale, accuracy and reproducibility of experimentation while freeing scientists from repetitive manual tasks (Chao *et al.*, 2017; Hillson *et al.*, 2019). To date, they have mainly been employed in industrialised biotechnology, however, as many publicly‐funded biofoundries have recently been established, their use in academically driven projects is set to grow (Hillson *et al.*, 2019).

Microfluidic platforms offer alternative avenues to automation and miniaturisation, often in combination with single‐cell experimentation. These approaches are sometimes referred to as ‘lab‐on‐a‐chip’ because they enable an ‘end‐to‐end’ laboratory workflow comprising DNA assembly, delivery to cells, analysis of gene expression and phenotypic screening, all on an integrated microfluidic platform (Linshiz *et al.*, 2016). Such approaches have mainly been applied in unicellular microorganisms. However, individual plant protoplasts can be captured within microfluidic chambers or spherical hydrogel beads (Nezhad, 2014; Grasso & Lintilhac, 2016) and microfluidic phenotyping of protoplasts has recently been reported (Yu *et al.*, 2018). The development of on‐chip genetic manipulation and characterisation of plant cells is therefore technologically possible in the near‐term.

The large‐scale experiments enabled by standardisation and automation necessitates the use of computational and statistical approaches in the design process. Firstly, these can be used to facilitate and document the design of, for example, synthetic genetic circuits. Secondly, they can be applied to define which circuits need to be built and tested by refining the experimental design. To aid the former, a number of CAD packages for designing complex genetic circuits from standardised parts have been developed. Gene Designer and BioStudio were developed to facilitate the design and construction of synthetic yeast chromosomes (Richardson *et al.*, 2010, 2017); GenoCAD, which has been applied to the design of synthetic genetic constructs for plants, exemplified the use of formal language for enabling biological design (Czar *et al.*, 2009; Coll *et al.*, 2015); j5 provides a drag and drop interface for arranging genetic parts into complex designs (Hillson *et al.*, 2012); and Cello automates design by drawing on libraries of genetic Boolean logic gates, molecular devices that implement a logical function to produce a binary output dependent on one or more binary inputs (Nielsen *et al.*, 2016). Typically, a given design process will result in numerous combinations for which, even aided by automation, testing would be inordinately laborious and expensive. It is difficult to predict how biological parts, even DNA parts, will behave in new combinations because interactions with other parts influence their properties and behaviours. While biologists typically learn to vary a single parameter at a time, the combined influence of factors may be nonlinear, therefore obtaining an optimal configuration requires optimising multiple factors in combination. The use of statistical experiment design, also known as design of experiments (DoE), has therefore been applied in synthetic biology to understand the relative importance and influence of multiple factors on the desired outcome while reducing the overall number of experiments (Exley *et al.*, 2019). DoE mathematically defines relationships between influencing factors and applies statistics to infer an experimental design by which only the necessary combinations (rather than every possible combination) need to be tested to obtain the equation. Following a history of use in bioprocess engineering it has been adopted into synthetic biology to, for example, optimise metabolic pathways (Brown *et al.*, 2018; Exley *et al.*, 2019).

## III. Engineering gene regulation

The selective and precise control of gene expression is essential for programming cells to perform new tasks. Although protein and metabolite levels may be controlled at numerous levels, information flow from synthetic genetic circuits is initiated by transcription. Consequently, there has been substantial effort focussed on engineering regulatory tools for controlling transcription. For many applications, it is considered desirable for synthetic regulatory elements to be orthogonal to the host cell, responding only to components encoded within the circuits or to externally provided inputs such as chemical ligands. In reality, while orthogonal components can reduce the effects of changes in the cellular environment on circuit performance, the synthetic system is heavily connected to the molecular and metabolic network of the cell and thus orthogonality may be considered as metaphorical rather than literal (de Lorenzo, 2011). In plants, orthogonality is sometimes undesirable as there is as much interest in the rational re‐engineering of existing genetic networks as there is in designing synthetic networks. However, the incomplete characterisation of the regulatory elements in any plant species, renders it challenging to rationally design synthetic elements that respond predictably to specific endogenous factors. To date, therefore, most efforts have focussed on the design of synthetic elements that respond specifically to external signals.

**Fig. 5:**
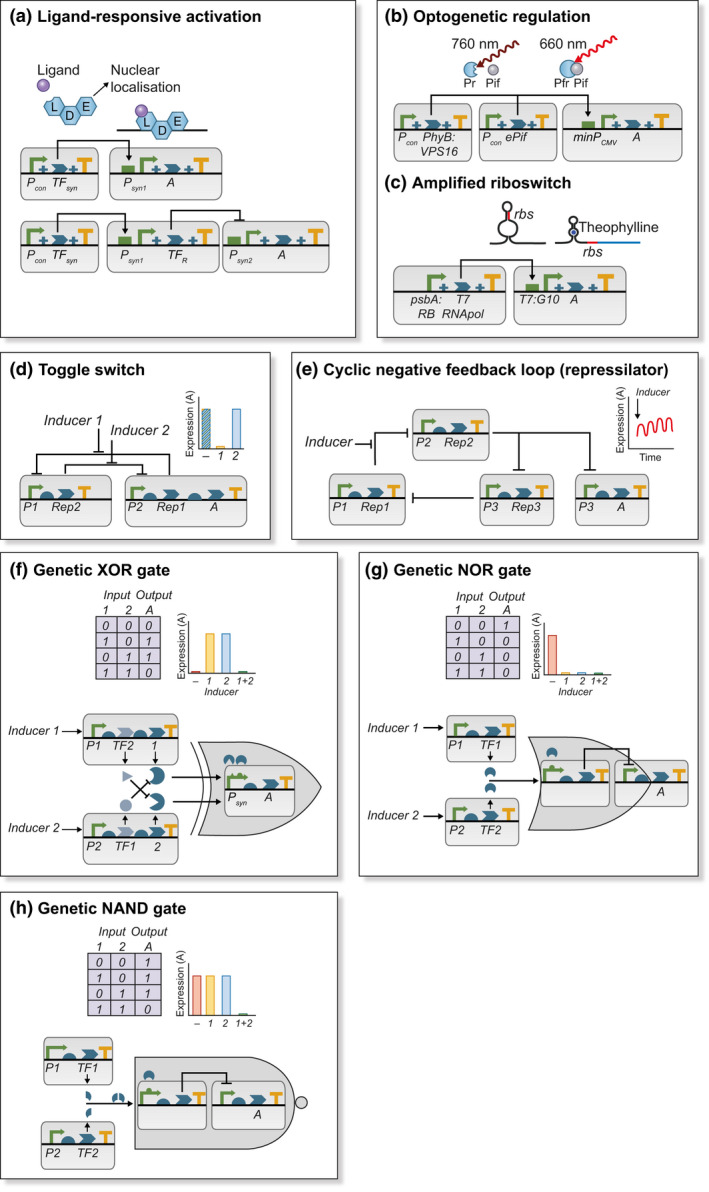
Synthetic genetic switches and circuits for regulating gene expression. (a) In simple ligand‐responsive switch, synthetic transcription factors (TF_syn_) formed from ligand‐binding (L), DNA‐binding (D) and effector (E) domains are expressed from a constitutive promoter (P_con_). In the presence of the ligand, TF_syn_ localises to the nucleus and binds to a synthetic promoter P_syn1_ activating expression of target gene ‘A’. The switch can be converted to a repressor by fusing P_syn1_ to a repressor of transcription (TF_R_) that binds to cognate motifs in P_syn2_. (b) In far‐red light (760 nm) there is no expression from the minimal promoter, minP_CMV_. In red light (660 nm), phyB, C‐terminally fused to the VP16 transactivation domain, interacts with an engineered phytochrome interacting factor (ePIF) and binds to cognate sites in minP_CMV_ inducing expression of ‘A’. (c) The ribosomal binding site (rbs) is located within a theophylline‐responsive riboswitch in the transcript of T7 RNA polymerase. In the presence of the ligand, translation is possible. This subsequently activates expression of target gene ‘A’ from a synthetic T7:G10 promoter. (d) In a genetic toggle switch, repressor of transcription 1 (Rep1) inhibits transcription from promoter 1 (P1) and is induced by inducer 1. Repressor of transcription 2 (Rep2) inhibits transcription from promoter 2 (P2) and is induced by inducer 2. Thus, in the presence of inducer 1, P1 is de‐repressed, resulting in repression of P2 (OFF) and, in the presence of inducer 2, P2 is de‐repressed resulting in repression of P1 (ON). In the absence of any inducer, the switch may stabilise at either setting. (e) A cyclic negative feedback loop or ‘repressilator’ can be used to produce oscillatory expression of a target gene ‘A’. The exact amplitude and period of oscillations are dependent on the repressor‐promoter affinity and stability of the repressors. (f–h) Boolean logic can be genetically encoded to enable output (expression of target gene ‘A’) to be dependent on the presence of multiple inputs (inducers). (f) In this realisation of an XOR gate, expression is induced by either transcription factor 1 (TF1) or 2 (TF2) but not both. This is achieved by co‐expression of an interacting protein for the competing TF preventing activation of P_syn_. (g) In the NOR gate, either TF can activate expression of a transcriptional repressor thus repressing ‘A’. (h) In the NAND gate, repression of the transcriptional repressor requires induction of both parts of a heterodimeric TF. The behaviour of each circuit is shown as a truth table and as idealised gene expression of ‘A’.

User control over gene expression in plants has long been achieved using ligand‐responsive systems. Systems that respond to applications of, for example, estradiol (Zuo *et al.*, 2000) typically comprise a constitutively expressed synthetic transcription factor used in combination with a synthetic promoter with cognate binding sites fused to the coding sequence of interest (Fig. 5a). In the presence of the specific chemical ligand, the synthetic transcription factor is activated, binding to the synthetic promoter. The basic components of such systems have also been engineered to construct more complex circuits that allow, for example, concurrent activation and repression (Schaumberg *et al.*, 2015). However, a drawback of chemical ligands is that they do not easily allow for spatiotemporal control. By contrast, light‐dependent protein interactions provide the potential for on‐demand deployment in a restricted number of cells using a focussed light‐source. So‐called optogenetic switches make use of photoreceptors from plants and bacteria that undergo light‐regulated conformational changes that enable or disable interactions with transcription factors or DNA‐binding domains (for review see Andres *et al.*, 2019). Some different optogenetic switches have been engineered to enable the expression of target genes to be controlled by the application of light of specific wavelengths. Their use in plants, which generally requires daily exposure to broad‐wavelength light, is complicated not only by the inability to achieve normal growth and development in restricted light, but also because plants contain photoreceptors and light‐sensitive pigments, which it may be undesirable to cross‐activate. Nevertheless, optogenetic control has been demonstrated in plants. A phytochrome‐based system activated by red light and inactivated by far‐red light was deployed in Arabidopsis, *Nicotiana tabacum* and *Physcomitrella patens* by supplementing normal lighting with low intensities of far‐red light to keep the system repressed (Müller *et al.*, 2014; Ochoa‐Fernandez *et al.*, 2016; Fig. 5b). A system employing the green‐light‐inducible bacterial photoreceptor, CarH was deployed in Arabidopsis protoplasts, reasoning that green light does not produce physiologically active signalling responses of relevance (Chatelle *et al.*, 2018).

The ability to programme DNA binding domains to recognise sequences of interest enabled control over the expression levels of endogenous as well as introduced genes. The ability to reprogramme Transcription Activator Like Effectors, proteins secreted into plants by bacterial pathogens to modulate the expression of genes that facilitate infection, provided tools to control the expression of endogenous plant genes as well as transgenes driven by synthetic promoters with cognate binding sites (Brückner *et al.*, 2015). RNA‐guided DNA nucleases such as Cas9 and Cas12a from bacterial Clustered Regularly Interspaced Short Palindromic repeats (CRISPR) systems for adaptive immunity have also been repurposed as synthetic genetic regulators, applied to control the expression of endogenous plant genes (e.g. see Li *et al.*, 2017). A Cas9‐based system for hormone‐responsive gene expression was engineered by fusing an auxin‐induced degron to a deactivated Cas9 (Khakhar *et al.*, 2018).

The expression of engineered genes can also be controlled post‐transcriptionally. Micro‐interfering RNAs (miRNAs) play an important role in natural gene regulatory circuits and artificial miRNAs (amiRNA) are widely used for silencing target genes (Ossowski *et al.*, 2008). More recently, miRNAs have been used to develop synthetic genetic circuits in *Chlamydomonas reinhardtii* (Navarro & Baulcombe, 2019). Optogenetic regulation of amiRNAs has also been used to control hydrogen production in this species (Wang *et al.*, 2017). Another set of tools employed for post‐transcriptional regulation are riboswitches. These are RNA sensors found in mRNAs across bacterial and eukaryotic lineages that are able to bind specific small molecules. They are typically found encoded in untranslated regions (UTR) and introns and modify expression by altering transcription, translation or splicing. Riboswitches have been extensively deployed for controlling gene expression in microbes, for example in the design of genetic Boolean logic gates (Maung & Smolke, 2008). A thiamine pyrophosphate responsive riboswitch system from *C. reinhardtii* was re‐engineered into an inducible repressor for studying the function of essential genes (Ramundo *et al.*, 2013) and also as a biosensor (Bocobza & Aharoni, 2014). An efficient theophylline‐responsive riboswitch was constructed by encoding T7 expression elements into the synthetic promoter used to drive the gene of interest, the expression of which was regulated via a riboswitch‐controlled T7 RNA polymerase (Emadpour *et al.*, 2015; Fig. 5c). This switch enabled the yield of HIV‐1 Nef antigen to be tripled in transplastomic *N. tabacum* (Emadpour *et al.*, 2015).

Tightly regulated and characterised switches are essential components of synthetic genetic circuits. While few extensive synthetic networks have been constructed in plants, in nonplant systems the synthetic biology field has progressed to employing such switches in complex regulatory networks. This has been facilitated by the application of mathematical and engineering approaches such as control theory. The early field of synthetic biology was greatly motivated by two landmark papers that described the function and behaviour of two simple genetic circuits formally and mathematically. These were a genetic toggle switch that can be flipped between stable states by transient application of externally supplied signals (Gardner *et al.*, 2000; Fig. 5d) and the repressilator, a genetic circuit from which output continuously oscillates (Elowitz & Leibier, 2000; Fig. 5e). This led to the development of a complete suite of synthetic genetic Boolean logic gates (Tamsir *et al.*, 2011; examples in Fig. 5f–h) as well as to memory devices that retain information of past events (Siuti *et al.*, 2013). The emerging field of cybergenetics combines control theory and cybernetics for the rational design of synthetic gene circuits that function as dynamic genetic control systems, predicting where feedback loops can be employed to improve features such as robustness, or to track cellular conditions and to adapt to changes in the environment. This has furthered the design of synthetic circuits able to respond to unpredictable changes in cellular environment, for example, the development of synthetic insulin‐sensitive transcription‐control devices (Ye *et al.*, 2017). Despite these impressive achievements, differences between the behaviours of designed and naturally evolved genetic circuits are inevitably encountered. Advocates of the engineering approach posit that the process of rewiring and reconstructing regulatory networks contributes to the understanding of how phenotypes emerge from network functions (Bashor & Collins, 2018).

## IV. Engineering proteins

The expression of synthetic genetic circuits can also be controlled at the post‐translational level. For example, Faden *et al*. (2016) developed an N terminal tag that triggers protein degradation at elevated temperatures, enabling heat‐activated control of protein levels in Arabidopsis. In common with natural oscillatory rhythms such as the circadian clock, the exact oscillatory behaviour of the synthetic repressilator (Fig. 5e) described in the previous section is a function of the relative stability of the regulatory proteins (Elowitz & Leibier, 2000). Protein engineering is a long‐established research area, driven as much by industrial requirements for highly stable enzymes able to function at expanded ranges of temperature and pH as by fundamental investigations of protein function.

Protein engineering used to be limited to the modification of naturally occurring protein sequences via substitutions, insertions or deletions of amino acids, typically by minor alterations of the coding sequence. However, advances in DNA assembly have empowered domain shuffling through combinatorial assembly of standardised DNA parts (Engler *et al.*, 2009; Fujishima *et al.*, 2015), and computational modelling has enabled the *de novo* design of novel proteins guided by structure and, more recently, by machine learning (Huang *et al.*, 2016; Bedbrook *et al.*, 2017; Alley *et al.*, 2019). The diverse range of protein engineering techniques can be roughly grouped into three categories: the design of synthetic proteins comprised of fused functional domains of different origins; rational design, usually structure‐guided, of naturally occurring proteins and *de novo* protein design; and methods for sequence diversification including error‐prone PCR, site‐saturation mutagenesis and domain shuffling. This final category also includes the use of directed evolution, which alternates between genetic diversification and selection of variants with the desired functional improvements.

Synthetic proteins with multifunctional properties formed by fusing multiple domains have been widely deployed in plants, notably, but not limited to, synthetic regulators (e.g. Zuo *et al.*, 2000; Khakhar *et al.*, 2018). There have also been many impressive examples of structure‐guided engineering of plant proteins to, for example, enable the synthesis of novel metabolites (Bhan *et al.*, 2015); alter the photochemistry or thermal stability of chromoproteins (Zhang *et al.*, 2013); uncouple the activities of multifunctional enzymes (Shivhare *et al.*, 2019); or widen the recognition profile of immune receptors (De La Concepcion *et al.*, 2019). In nonplant systems, computational methods are advancing structure‐guided engineering (Gainza‐Cirauqui & Correia, 2018). These have been used to increase the substrate‐specificity of enzymes (Mak *et al.*, 2015), engineer protein–protein interactions (Rose *et al.*, 2017) and to design protein scaffolds with specified curvatures (Park *et al.*, 2015b). Recently, machine‐leaning approaches have been applied to overcome the relative scarcity of structural data for many protein types (Bedbrook *et al.*, 2017; Alley *et al.*, 2019). Computationally designed protein domains have also been deployed in plants: an artificial signalling pathway used in Arabidopsis used a bacterial periplasmic binding protein engineered using computational analysis to predict the amino‐acid sequences that would form the complementary surface between the protein and a new target ligand, trinitrotoluene (Looger *et al.*, 2003). Plant scientists fused this engineered ligand‐binding domain to a signal peptide from a plant receptor‐like and a modified bacterial histidine kinase to create a synthetic receptor kinase (Antunes *et al.*, 2011). Similarly, ligand‐binding domains for digoxigenin (Tinberg *et al.*, 2013) and fentanyl (Bick *et al.*, 2017) were designed using computational methods to optimise hydrogen bonds between the ligand and receptor. These domains were subsequently fused to destabilised fluorescent proteins and transcriptional activation domains to create plant‐based biosensors (Feng *et al.*, 2015; Bick *et al.*, 2017).

Interestingly, the directed evolution of proteins is alternately considered as a flagship technology of synthetic biology (Cobb *et al.*, 2013), or, completely at odds with a field committed to improving the predictability of rational design (Davies, 2019). Methods for directed evolution were established before computational methods to predict the impact of amino‐acid substitutions (particularly multiple mutations) on protein properties had begun to show promise. While predictive abilities are still limited, computational methods and random mutations have recently been combined for machine‐learning‐guided directed evolution. This has been demonstrated in bacteria with fluorescent proteins (Saito *et al.*, 2018) and also to produce enzymes able to perform new‐to‐nature chemical transformations (Wu *et al.*, 2019). In plants, long life cycles, low transformation efficiency and difficulties in high‐throughput phenotyping can limit large‐scale mutagenesis and screening. However, a plant receptor kinase with expanded recognition of a microbe‐associated molecular pattern was obtained using *in vitro* evolution followed by a functional screen in Arabidopsis (Helft *et al.*, 2016). RuBisCO was engineered using directed evolution in *Rhodobacter capsulatus* (Smith & Tabita, 2003) and *Escherichia coli* (Parikh *et al.*, 2006). More recently, a ‘Cas9‐mediated directed evolution approach’ was demonstrated in rice by deploying 119 sgRNAs to identify protein variants that confer resistance to a splicing inhibitor (Butt *et al.*, 2019).

## V. Engineering metabolism

Metabolic engineering is generally executed by the insertion of synthetic genetic circuits or by perturbing the expression levels or function of endogenous genes using the approaches described in the previous sections. Before any intervention, however, targets must be identified and an engineering strategy must be established. The application of predictive metabolic modelling, employing information on flux through networks of interdependent enzyme‐catalysed chemical reactions, has long been applied in metabolic biology (Edwards *et al.*, 2001; Segrè *et al.*, 2002). It is therefore unsurprising that, by combining these methods with new tools for engineering, synthetic biology has been able to make such rapid progress in metabolic engineering.

In microbial systems, a significant focus of metabolic engineering has been the production of natural products, frequently of plant origin, of industrial and pharmaceutical interest (Liu *et al.*, 2017; Cravens *et al.*, 2019). The availability of numerous characterised genetic elements and tools for some bacterial species mean the construction and comparison of multiple configurations of heterologous genetic pathways to optimise production is relatively straightforward. Nevertheless, even when expression of all heterologous proteins is successful, product yield may be modest without the introduction of, what can be numerous, genetic modifications to redirect flux towards the molecule of interest (Nielsen & Keasling, 2016). This process is intrinsically difficult because the processes that control metabolic flux are highly regulated. Hence, genome‐scale metabolic models have been used to predict the influence of perturbations and substantially increase yields (Nielsen & Keasling, 2016; Gu *et al.*, 2019).

Plant hosts offer the distinct advantages of multiple cellular compartments and the ability to more easily express proteins that are challenging in some microbes. However, the complexity of plant specialised metabolism makes predictive engineering a hugely difficult task. While several small molecules, particularly terpenoids from other plant species, have been heterologously expressed in species of *Nicotiana* (Hasan *et al.*, 2014; Vasilev *et al.*, 2014; Cankar *et al.*, 2015), only a few studies have sought to do more than simply express the heterologous enzymes. Competition with other pathways can, to a point, be negated by transient expression in mature tissues (Reed & Osbourn, 2018) or by limiting expression to specific organs such as seeds and fruits (Li *et al.*, 2018). In these cases, the heterologous pathway is not competing for carbon with pathways essential for growth and development and, provided the concentrations of metabolic precursors and the heterologous enzymes are sufficiently abundant, substantial yields can be obtained without substantial engineering of upstream or competing pathways. While transient expression has been hugely successful for obtaining high yields of some classes of molecules, for others, the rich endogenous metabolism of the host plants is problematic; several studies have reported the derivatisation of pathway intermediates and final products (Brückner & Tissier, 2013; Liu *et al.*, 2014; Dong *et al.*, 2016). Other studies have stably integrated transgenes to express heterologous pathways, attempting to increase the pool of precursor molecules by overexpression of upstream biosynthetic pathway genes (Brückner & Tissier, 2013) or by silencing enzyme‐encoding genes from competing pathways (Cankar *et al.*, 2015). Impressive increases in yields of artemisinic acid in *N. tabacum* were achieved by combinatorial testing of all enzymes known to affect flux into the pathway (Fuentes *et al.*, 2016).

Applications of model‐informed engineering of primary metabolism to improve agricultural traits have been remarkably successful. Computer modelling was applied to investigate how differences in expression of alternative photorespiratory pathways for recapturing unproductive by‐products of photosynthesis might affect flux. This informed an engineering strategy for expression of an optimal synthetic glycolate metabolic pathway that significantly increased yield, the implementation of which was facilitated by the use of interoperable standardised parts (South *et al.*, 2019). Modelling approaches are now being widely applied to identify further engineering strategies for crop improvement: Models of photosynthesis were used to evaluate the efficacy of introducing C_4_ photosynthesis into rice (Bellasio, 2017; Bellasio & Farquhar, 2019) concluding that, even if C_4_ photosynthesis is introduced without changing morphological features such as vein spacing, this may provide substantial yield benefits (Ermakova *et al.*, 2020). In recent work, flux control analysis was used to identify soybean enzymes in which changes would best facilitate adaptation to rising CO_2_ (Kannan *et al.*, 2019). By linking gene regulatory networks through protein concentration to the model, transcription factors (TFs) were identified for which manipulation of gene expression levels would most likely improve photosynthesis (Kannan *et al.*, 2019). The energy costs associated with high‐turnover proteins were predicted by using crop growth simulations identifying a small number of proteins that if turnover was reduced, potentially by protein engineering, accumulation of biomass would be positively affected (Hanson *et al.*, 2018). A wider investigation identified additional engineering strategies that could increase biomass by reducing respiratory carbon loss (Amthor *et al.*, 2019). Although the realisation of any of these designs will require significant effort, the availability of new tools for designing and manipulating plant genes combined with predictive modelling is revolutionising plant metabolic engineering.

## VI. Emerging research areas

**Fig. 6:**
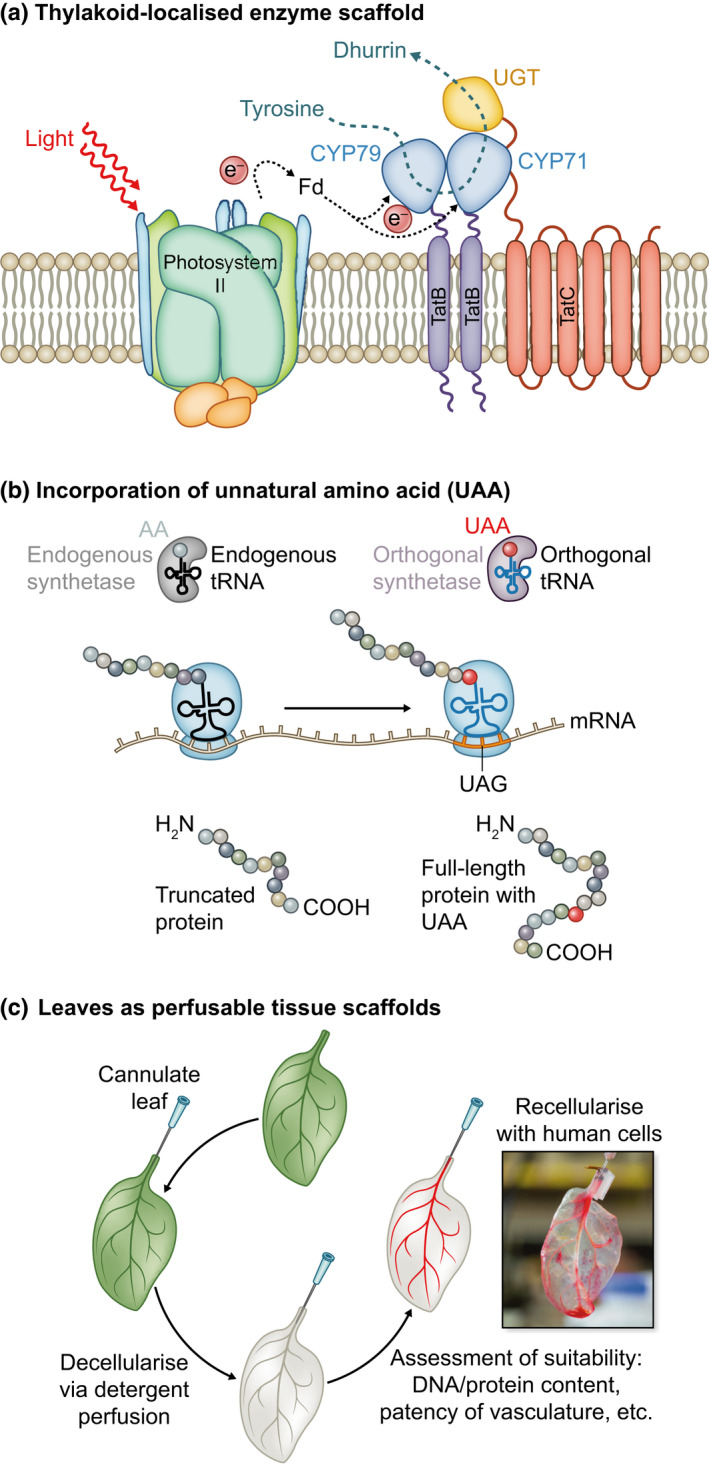
Prototypes of emerging areas in plant synthetic biology. (a) Scaffolding of a UDP glucosyl transferase and two cytochrome P450s (CYP97 and CYP 71) in the thylakoid membrane by fusion to ΔpH‐dependent twin‐arginine translocation (TatB and TatC) transmembrane proteins. Ferredoxin (fd) serves as an electron donor. Adapted from Henriques de Jesus *et al*. (2017). (b) Unnatural amino acids (UAA) are incorporated into heterologous proteins by expression of orthologous tRNA and tRNA‐synthetase pairs. In the system deployed in Arabidopsis, these recognise the rare UAG amber stop codon. When the UAA is supplied it is incorporated into the polypeptide chain resulting in the production of a full‐length protein. Adapted from Li *et al*. (2013). (c) Leaves used in engineering human tissues are decellularised with detergents. The vascular system is then recellularised with specific human cell types. These can be supplied with nutrients through the vascular system, enabling them to form tissues with novel structures. Adapted from Gershlak *et al*. (2017).

Some new and exciting areas of research have recently been exemplified in plants. For example, the use of synthetic complexes for metabolic engineering is a growing field of research. Synthetic microcompartments are valued in biomanufacturing for segregating heterologous metabolic pathways from the wider cellular environment. Bacterial microcompartments (BMCs) have been engineered into bacterial, yeast and mammalian cells (Kerfeld *et al.*, 2018; Lau *et al.*, 2018). Efforts have been extended to plants through the engineering of carboxysomes in chloroplasts with the aim of enhancing CO_2_ fixation (Gonzalez‐Esquer *et al.*, 2016). Genes for alpha‐ and beta‐carboxysomes have been successfully engineered into the chloroplast genomes of *Nicotiana* species (Lin *et al.*, 2014; Long *et al.*, 2018). In addition, plant peroxisomes have been utilised for the production of polyhydroxyalkanoates, which can be used as biodegradable plastics, (Schenk *et al.*, 2014). As well as separating toxic or reactive intermediates from the cell, an advantage of microcompartments is co‐localisation of pathway enzymes. An alternative method to achieving this is the use of molecular scaffolds to which enzymes can be tethered. In microbes, the use of synthetic scaffolds has been demonstrated to improve molecular flux through heterologous pathways (Dueber *et al.*, 2009). Recently, synthetic scaffolding of three enzymes using TatB and TatC proteins to anchor the pathway in the thylakoid membrane, was shown to result in a five‐fold increase in yield of the cyanogenic glucoside, dhurrin (Fig. 6a; Henriques de Jesus *et al.*, 2017).

Another synthetic biology research area that has been widely applied in nonplant systems is the introduction of nonnatural genetic components. Unnatural amino acids (UAAs), those beyond the canonical 20 used by the majority of organisms, have been used to control post‐transcriptional modifications and to enable site specific labelling for visualisation in living cells, as well for the production of antibody drug‐conjugates (Wang *et al.*, 2001; Neumann‐Staubitz & Neumann, 2016; Nödling *et al.*, 2019). In plants, a lysine analogue, *N*‐ε‐acryllysine, was incorporated into a fluorescent protein in Arabidopsis by co‐expression of an orthogonal tRNA that recognises UAA, a corresponding tRNA synthetase and a GFP coding sequence with a premature UAA stop codon. A full‐length fluorescent protein was only obtained when the UAA was successfully incorporated (Li *et al.*, 2013; Fig. 6b).

Particularly distant to the dominant research areas of plant biology are biomimetic biological systems. However, the development of such systems have long been a focus of synthetic biology. In particular, protocells and genetic circuit‐containing synthetic minimal cells (synells) are used to investigate intracellular metabolic reactions and to study biological behaviours (Miller & Gulbis, 2015; Adamala *et al.*, 2017). Although such biomimetic systems can hardly be classed into a kingdom of life, of relevance to plants is a photosynthetic protocellular system based on a plant‐derived photosystem II (PSII) and bacteria‐derived proteorhodopsin (PR) (Lee *et al.*, 2018). Like plant chloroplasts, the photosynthetic artificial organelle functioned as an energy module. Such systems may provide novel opportunities for plant biologists as simplified platforms for prototyping and for detailed functional characterisations.

The engineering of biologically derived materials into useful products sits at the cusp of synthetic biology and biomaterials. As synthetic biology approaches are being used to engineer biomolecules and materials, convergence could lead to new developments in the control of tissue organisation, the understanding of which is a major goal in fundamental biology and essential for progress in regenerative biology (Keating & Young, 2019). Plant‐derived products, particularly celluloses, are already used for a wide number of so‐called ‘smart’ materials such as electroactive polymers and hydrogels (Rebouillat & Pla, 2019). Impressively, plants are now being used to engineer human tissues. Plant‐based scaffolds have many of the desirable features for synthetic tissues: biodegradability, pliability, low cost and a vasculature useful for the delivery of nutrients. In a recent proof‐of‐concept, ‘decellularised’ plant tissues were demonstrated to function as prevascularised scaffolds for tissue engineering, which human endothelial cells were able to colonise (Fig. 6c; Gershlak *et al.*, 2017).

Synthetic genomics, the bottom‐up redesign of genomes, is often considered as the ultimate exemplification of synthetic biology. The first genome‐scale DNA assembly project was completed in 2008 (Gibson *et al.*, 2008), followed rapidly by the synthesis and assembly of a mouse mitochondrial genome (Gibson *et al.*, 2010), synthetic yeast chromosomes (Dymond *et al.*, 2011; Richardson *et al.*, 2017), a minimised *Mycoplasma genitalium* genome with 52 fewer genes (Hutchison *et al.*, 2016), and a synthetic *E. coli* genome that uses just 59 codons to encode 20 amino acids (Fredens *et al.*, 2019). These projects have demonstrated the ability to design, build, validate and characterise large DNA assemblies. There is enthusiasm for larger eukaryotic chromosomes, including plants, within Genome Project Write (GP‐Write), the consortium of scientists focussed on synthetic genomics. However, several technical bottlenecks in design, synthesis and construction need to be overcome (Ostrov *et al.*, 2019). At the time of writing, these are being explored through the execution of smaller projects, including the assembly of a synthetic plant chloroplast genome (N. Stewart, pers. comm.).

These emerging research areas are developing exciting opportunities for plant science. Alongside advances in knowledge and novel technologies, these projects also provide the potential of new roles for plants in human life and society.

## VII. Applications and prospects

The ability to engineer predictably is hampered by the unknown. Iterative progressions of design–build–test–learn cycles are useful not only for optimising the performance of synthetic systems but also for improving models of biology. Synthetic biology approaches are therefore as relevant to fundamental plant biology as they are to plant biotechnology. However, many of the approaches discussed above remain challenging in long‐lived plant species with low transformation efficiencies. The use of modelling and streamlining experiments through, for example, the application of DoE, can be used to focus engineering efforts. However, more tractable species are better suited for complex manipulations. The numerous genome‐scale datasets and resources available for Arabidopsis underpin its importance in plant science, but simpler species such as the liverwort, *Marchantia polymorpha*, offer advantages for the systems‐scale experiments that aid synthetic biology. Able to complete its lifecycle within a Petri dish, it is possible to track physiological and morphogenetic changes through the entire life cycle. Comparatively low gene numbers mean that functional studies of gene families, reconstruction of transcriptional networks, and the development of genome‐scale models are simplified (Boehm *et al.*, 2017). Although it may be more difficult to directly apply learning into crops, simpler systems are ideal for rapid prototyping of synthetic genetic circuits and demonstrating new tools and approaches. Synthetic biology provides the exciting possibility of using rational design to explore beyond the confines of the current natural world. Either by using genetic information from extinct lineages to inform new designs, or by exploring the function of DNA and amino‐acid sequences that have never evolved, synthetic biology can help to define and probe the limits of possible phenotypic space for small molecules (Klein *et al.*, 2014), proteins (Woolfson *et al.*, 2015), and even tissues (Toda *et al.*, 2018). While such approaches have yet to be seriously applied in plant systems, they are already being discussed (Wurtzel *et al.*, 2019).

Synthetic biology approaches are being applied to numerous applications of plant biotechnology. In this review there are examples of how synthetic biology is being applied to increase crop yields (South *et al.*, 2019) and to modify plant responses to environmental signals (Vaidya *et al.*, 2019). Europe remains a challenging market for biotech crops and conversations about public acceptance of genetically modified foods have continued through the 25 yr since the first products reached market. Despite this, biotech crops are now grown on 191 million hectares worldwide and, in the last year, several new traits have reached market that mainly provide benefits to consumers and thus may move the conversation beyond herbicide tolerance and pest‐resistance (ISAAA, 2018). Nevertheless, the route to market for many promising biotech crops is likely to be challenging. Others have proposed that nonfood applications of biotechnology such as biofuels might find wider public acceptance (Fesenko & Edwards, 2014). Certainly, there are many plant synthetic biology projects that are not intended for consumption including the development of plants as sentinels and biosensors (Bick *et al.*, 2017), as sources of medical biomaterials (Gershlak *et al.*, 2017), and as platforms for biomanufacturing (Fuentes *et al.*, 2016; Henriques de Jesus *et al.*, 2017; Reed *et al.*, 2017). Plant‐based biomanufacturing of human therapies has been discussed for decades with numerous proofs of concepts being published. Although there are still high barriers to market entry, in the last decade there have been decisive steps forward with many plant‐made products progressing to clinical trials and the marketing of plant‐produced taliglucerase (Eleysio™; Buyel, 2019).

The strength of synthetic biology lies in its intellectual diversity. While the majority of presenters at a typical plant science meeting will have trained as biologists, at synthetic biology meetings mathematicians, computer scientists, engineers, biologists, chemists and physicists will all be present in varying proportions. Arguably, the most thrilling achievements have been made at the overlaps of traditional disciplines (Cameron *et al.*, 2014). Plant synthetic biology is still several steps behind microbial and mammalian synthetic biology. However, the importance of plants as sources of food, materials and products and as a source of feedstocks for microbial growth is widely recognised. The potential of developing, for example, synthetic genetic networks, unnatural genetic systems and synthetic genomes for plants are clearly demonstrated by the impact of these advances in microbial and mammalian biology and biotechnology. Progress in plants is understandably hampered by low transformation efficiencies and comparatively long life cycles but it is also held back by a lack of comprehensive information about functional DNA elements (particularly regulatory elements), single‐cell expression data for multiple organs, and methods for directed evolution in plant cell cultures. As recently noted, plant biotechnology would particularly benefit from investment in infrastructures that facilitate large‐scale experimentation in plants, including biofoundries with plant‐workflows (Wurtzel *et al.*, 2019). Perhaps the most critical changes required to realise the potential of plant synthetic biology are a wider adoption of engineering principles and a greater integration of systems biology, computational modelling and machine learning into plant biology.
